# Complete genome sequence of *Peptostreptococcus porci* isolated from porcine endocarditis in Japan

**DOI:** 10.1128/mra.00201-24

**Published:** 2024-06-06

**Authors:** Masahiro Hayashi, Jun Yonetamari, Yoshinori Muto, Kaori Tanaka

**Affiliations:** 1 Institute for Glyco-core Research iGCORE, Gifu University, Gifu City, Gifu, Japan; 2 Division of Anaerobe Research, Life Science Research Center, Gifu University, Gifu City, Gifu, Japan; 3 Gifu University Center for Conservation of Microbial Genetic Resource, Gifu City, Gifu, Japan; 4 Division of Clinical Laboratory, Gifu University Hospital, Gifu City, Gifu, Japan; 5 United Graduate School of Drug Discovery and Medical Information Sciences, Gifu University, Gifu City, Gifu, Japan; University of Maryland School of Medicine, Baltimore, Maryland, USA

**Keywords:** *Peptostreptococcus porci*, genomes

## Abstract

*Peptostreptococcus porci* is a recently described bacterium belonging to the *Peptostreptococcaceae* family, which was isolated in 2016 from pig intestine. Herein, we report the complete genome sequence of a clinical isolate of *P. porci* (GAI11004) obtained from porcine endocarditis in Japan. The genome contains a 2.4-Mb circular chromosome.

## ANNOUNCEMENT

Identification and investigation of livestock pathogens are important for biomedical research and the accumulation of agricultural knowledge. *Peptostreptococcus porci* is a Gram-positive, strictly anaerobic bacterium belonging to the Peptostreptococcaceae family; the type strain was isolated from a pig intestinal sample (1). However, the complete genome sequence is not available for any *P. porci* isolate. The strain used in this study (GAI11004) was isolated from porcine endocarditis. This strain was discovered at a slaughterhouse in Yamagata prefecture, Japan, in 2010. A festering foci-like nodule was observed on the tricuspid valve, some with fragile cauliflower-like lesions. The lesion was stamped directly onto the blood agar medium and cultured. The strain was isolated from the anaerobically cultured agar plate and submitted to our lab as a suspected *Peptostreptococcus* strain for further identification. The strain was subjected to API 20A, 32A (bioMérieux Japan Ltd., Tokyo, Japan) and 16S rRNA sequence analysis for identification. We confirmed the identification of this strain at the genus level (*Peptostreptococcus* spp.) but could not identify it at the species level. Therefore, the strain was stored at –80°C after several passages during the identification process.

Recently, the strain was cultured on Brucella HK agar (Kyokuto Pharmaceutical Industrial, Japan) supplemented with 5% laked sheep blood at 37°C for 48 h in anaerobic conditions (82% N_2_, 10% CO_2_, and 8% H_2_) and pelleted. Genomic DNA was extracted from the pellet using MonoFas (ANIMOS, Japan) for all genome analyses. Using BLASTN analysis of the PCR amplified full-length 16S rRNA gene, the strain was identified as *P. porci*, with a nucleotide sequence identity of 99.6%. The species was also confirmed with an Average Nucleotide Identity value of 96.3% against the *P. porci* type strain (ASM969581v1) using GTDB-tK (v.2.3.2) (2).

The entire genome of this isolate was sequenced using a combination of long-read sequencing by Nanopores [Oxford Nanopore Technologies (ONT), Tokyo, Japan] and short-read sequencing by DNBSEQ (MGITech Co. Ltd., Shenzhen, China) as previously described (3, 4). For long-read sequencing, a library was constructed using a ligation sequencing kit (SQK-LSK-109; ONT) without shearing. Small DNA fragments were removed using Short Read Eliminator (Pacific Bioscience of California Inc., USA), and sequencing was performed using a GridION X5 system (ONT) on a FLO-MIN106 flow cell. Long-read sequence data were based on Guppy v.5.0.12 (high-accuracy mode). The raw reads were subjected to trimming and quality filtering using NanoFilt v.2.7.1 (5) with the parameters “-l 1000 -q 10 --headcrop 50.” For short-read sequencing, the MGIEasy FS PCR Free DNA Library Prep Set (MGI Tech) was used for library construction. Subsequently, 2 × 150 bp paired-end sequencing was performed using the DNBSEQ-G400 platform (MGI Tech). Raw sequencing reads were processed using fastp v.0.20.1 (6) with the parameters “-q 30 -n 20 -t 1 -T 1.” The quality of the short reads was checked using fastp v.0.20.1 (6), and the mean read quality of the long reads was scored using NanoPlot 1.32.1. High-quality short reads (over 91% of bases > Q30 averaged) and long reads (mean read quality of 13.5) were assembled using Unicycler v.0.4.8 (7) with default settings. The assembly was rotated to start with the *dnaA* gene on the forward strand. The assembled contig graph was visualized using Bandage v.0.8.1 (8), and the integrity of the assembled genomic data was confirmed using blobtools v.1.1 (9).

The genome information is summarized in (Table 1). Quality assessment and genome statistics were computed using QUAST (v5.2.0) (10) and CheckM (v1.2.2) (11). According to CheckM, the genome assembled in this study was 100% complete and had 0.0% contamination. DDBJ Fast Annotation and Submission Tool (12) predicted 2,106 coding sequences, 15 ribosomal RNAs, and 60 transfer RNAs in total.

**TABLE 1:** Table 1 Information regarding the complete genome sequence of a *Peptostreptococcus porci* strain isolated from a porcine endocarditis.

	Strain name
Parameter	*P. porci* GAI11004
DNBSEQ sequencing^*a*^	
No. of reads	23,578,812
Size (kb)	3,536,822
Avg coverage (x)	1,482
DRA accession no.	DRR530170
ONT sequencing^*a*^	
No. of reads	696,934
Size (kb)	2,146,124
Avg read length (bp)	3,079
Avg coverage (x)	899
N50	4,420
DRA accession no.	DRR530169
Assembly	
Assembly N50 (bp)^*c*^	2,386,046
Estimated genome completeness (%)^*d*^	100.0
Estimated genome contamination (%)^*d*^	0.0
Genome structure	one chromosome
DDBJ/GenBank accession no.	AP029254 (GAI11004)
Genome size (bp)	2,386,046 (GAI11004)
GC content (%)	
(chromosome/ plasmid name)	33.6 (GAI11004)
No. of coding sequences^*b*^	2106
Number of rRNAs^*b*^	15
Number of tRNAs^*b*^	60
Number of CRISPRs^*b*^	2

^*a*^ DRA, DDBJ Sequence Read Archive.

^*b*^ DFAST, DDBJ Fast Annotation and Submission Tool.

^*c*^ Determined with Quast.

^*d*^ Determined with CheckM.

## Data Availability

The genome sequences of *P. porci* (GAI11004) strains from Japanese porcine endocarditis were deposited in DDBJ (https://www.ddbj.nig.ac.jp/index.html) with accession number AP029254. Raw sequence data for GAI11004 were deposited in the DDBJ/Sequence Read Archive under accession numbers DRR530169 and DRR530170.
